# Metagenomic applications in exploration and development of novel enzymes from nature: a review

**DOI:** 10.1186/s43141-020-00043-9

**Published:** 2020-08-04

**Authors:** Fitra Adi Prayogo, Anto Budiharjo, Hermin Pancasakti Kusumaningrum, Wijanarka Wijanarka, Agung Suprihadi, Nurhayati Nurhayati

**Affiliations:** 1 Department of Biology, Faculty of Science and Mathematics, Diponegoro University, Semarang City, 50275 Indonesia; 2 Biotechnology Study Program, Faculty of Science and Mathematics, Diponegoro University, Jl. Prof. Sudharto SH, Semarang, 50275 Indonesia; 3 Molecular and Applied Microbiology Laboratory, Center Central Laboratory of Research and Service - Diponegoro University, Jl. Prof. Sudharto SH, Semarang, 50275 Indonesia

**Keywords:** Metagenomics, Novel enzymes, Microbial community, Environmental DNA

## Abstract

**Background:**

Microbial community has an essential role in various fields, especially the industrial sector. Microbes produce metabolites in the form of enzymes, which are one of the essential compounds for industrial processes. Unfortunately, there are still numerous microbes that cannot be identified and cultivated because of the limitations of the culture-based method. The metagenomic approach is a solution for researchers to overcome these problems.

**The main body of the abstract:**

Metagenomics is a strategy used to analyze the genomes of microbial communities in the environment directly. Metagenomics application used to explore novel enzymes is essential because it allows researchers to obtain data on microbial diversity, reaching of 99% and various types of genes encoding an enzyme that has not yet been identified. Basic methods in metagenomics have been developed and are commonly used in various studies. A basic understanding of metagenomics for researchers is needed, especially young researchers to support the success of the research.

**Short conclusion:**

Therefore, this review was done in order to provide a deep understanding of metagenomics. It also discussed the application and basic methods of metagenomics in the exploration of novel enzymes, especially in the latest research. Several types of enzymes, such as cellulases, proteases, and lipases, which have been explored using metagenomics, were reviewed in this article.

## Background

The microorganism community from nature is the largest community that plays an essential role in the biogeochemical cycle on earth. Many microorganisms are also known to have a role in the development of the industry that exists today by the production of metabolites [1]. Enzymes are one of the microbial metabolites often used in the industrial processes.

Enzymes are biocatalyst compounds that can accelerate biochemical reactions used in various industries, such as textiles, paper, detergents, food, and beverages [2]. Various benefits of enzymes have attracted the attention of researchers to develop and explore enzymes from nature for further application in the industrial field. Unfortunately, there are still many types of microorganisms that are not identified yet and cannot be cultured in the growth media. The use of culture-based method only results in diversity data of less than 1% of the total microorganisms in the environment [3].

Metagenomics is a breakthrough for the weakness of culture-based method, which has sharply increased its application in recent years. In the metagenomics, DNA is directly extracted from the environment samples without culturing process in the laboratory. The use of DNA to analyze the diversity of microorganisms reveals a representative and comprehensive result [4, 5]. Metagenomics has been used in various fields of study, such as in the microbial communities of the human intestine [6], sugarcane bagasse waste [7], and hypersaline environment [8]. In addition to exploring the benefits of gene resources from nature, the existence of metagenomics studies can also increase knowledge about the relationships between microorganism communities in the biogeochemical cycle in nature.

The understanding of metagenomics needs to be reviewed further in order to deepen the insights of metagenomic studies. A thorough understanding of metagenomics and their application in research is expected to have an impact on increasing discoveries about the information of the microbial community and enzymes from nature. Therefore, this review is designed to discuss the application of metagenomics in the exploration of novel enzymes from nature. The focus of this review is to provide a deep understanding of metagenomics, basic method, and its utilization to enzyme exploration, especially in the latest research.

## Main text

### Metagenomics

Direct DNA extraction from the environment was started in 1985 by Pace and his team. However, the new term of metagenome emerged in 1998 by a researcher named Handelsman. Metagenomics is the study of genomes from microorganism communities in the environment [9, 10]. Other terms of metagenomics are community genomics, environmental genomics, and population genomics [4]. Metagenomics is a strategy used to analyze genomes acquired from the community of environmental microorganisms without culturing them [11]. This technique can read the diversity of microorganisms up to 99% of the total microorganisms in environmental samples [12]. Metagenomics becomes a new concept in microbiology studies, thus opening the horizons of researchers’ minds to discover new biochemical compounds that are available in nature and can be utilized in the biotechnology industry.

### The direction of metagenomics study

**Fig. 1:**
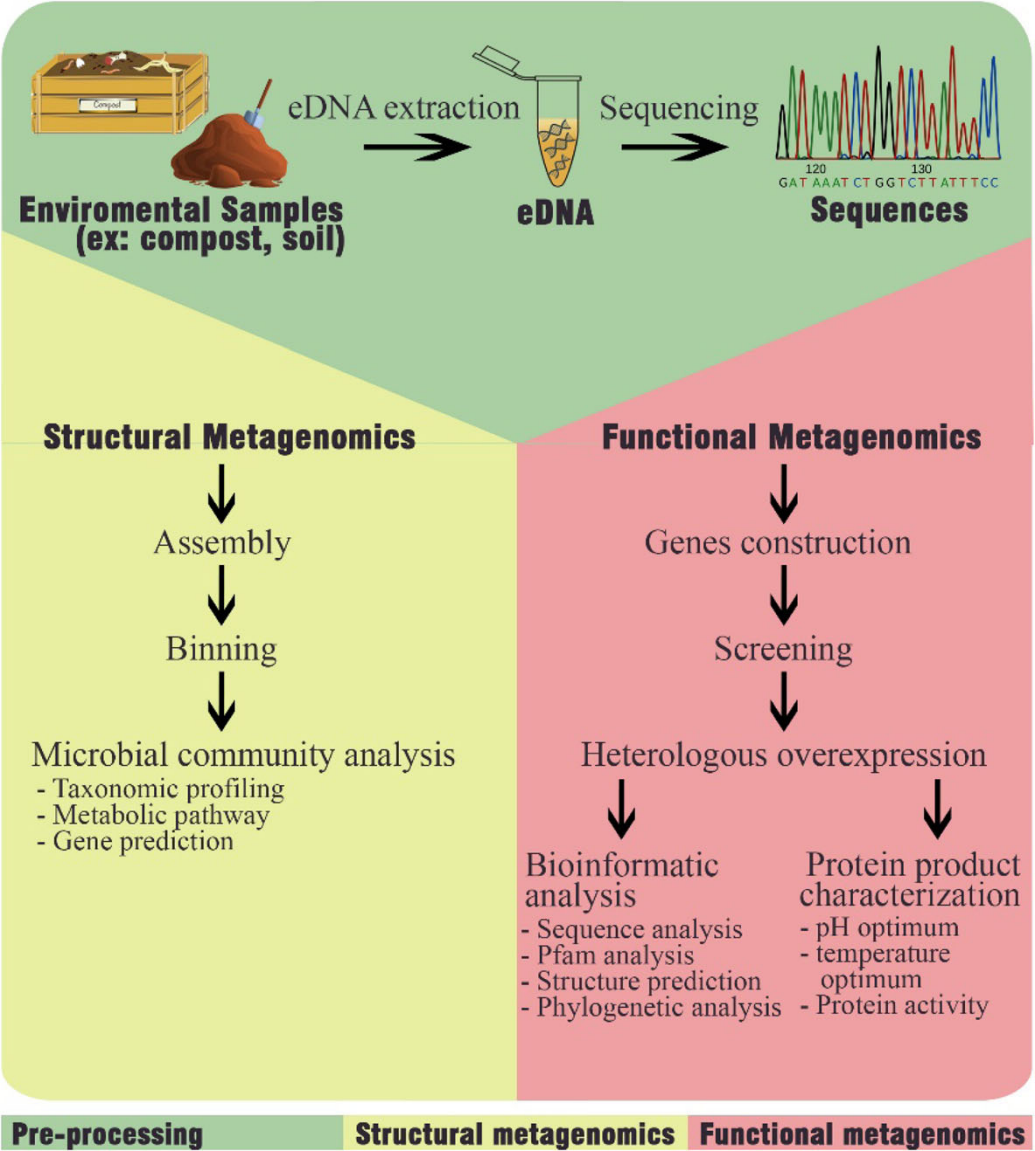
Framework for metagenomics with two primary studies, structural and functional metagenomics

Figure 1 shows the direction in a metagenomics study. Metagenomics is divided into two primary studies, namely, structural metagenomics and functional metagenomics [13]. Structural metagenomics is a study focused on the structure of microbial communities. The study of community structure focuses on understanding the relationships between individual components in building a community in an environment. Relationships between components in the community are essential information for studying ecology and biological functions [12]. Basic structural metagenomics methods consist of assembly, binning, and microbial community analysis such as taxonomic profiling, gene prediction, and metabolic pathways [3, 14].

Functional metagenomics is a study focused on the use of genes encoding a particular protein [12]. The study of functional metagenomics is a new challenge in exploring natural compounds that can be utilized in the biotechnology industry. Several basic methods in functional metagenomics are carried out to access the novel enzymes, like gene construction, screening, gene expression, and can be followed by bioinformatic analysis such as sequence, Pfam, structure prediction, and phylogenetic analysis and also protein product characterization such as optimum pH rate, optimum temperature rate, and protein activity analysis [10].

The two approaches, structural and functional metagenomics, are a strategy for the exploration of microorganism communities in ecology and biotechnology studies. This combination cannot be separated in metagenomic studies. Both are the basis of microbial ecological problems, namely, “What types of microorganisms exist in the environment?” Furthermore, “What is the function of these microorganisms in the environment?” [12].

### Microbial community as metagenomic research object

The microbial community is the largest community that plays an essential role in the biogeochemical cycle on the planet [1]. Microbial communities have the most diverse species on earth by forming 60% of the earth’s biomass [15]. The total number of microbes on earth is even predicted to reach 10^30^ [16]. The importance of the role of microbes in the “balance of life” on this planet makes us need to deepen understanding of the microbial community, so that ecosystem damage does not occur. Better ecosystem management and progress in bioprospection will be achieved with a fundamental understanding of interactions between microbial communities [1]. Microbial community with various biochemical reactions in it is a mystery that is still a question mark for researchers. The existence of metagenomics allows researchers to unravel the mysteries that are in it. The microbial community will be something interesting to continue to study.

Furthermore, the microbial community also has benefits in industrial processes. Various types of enzymes found in several publications come from the microbial community, such as cellulases [17], proteases [18], and esterases [19]. Enzymes originating from the microbial community have advantages for industry compared to animals and plants, such as being more stable, have high yields, and are easily engineered [20].

### Metagenomic sequencing technology

In the past, microbial analysis was done using pure culture. The use of pure culture by conventional isolation is a limiting factor in the analysis of environmental microbes. Microbial communities in the environment interact with each other to exchange nutrients, biochemical products, and chemical signals [21]. The presence of a microbial community complex system cannot be captured if it relies solely on a pure culture system.

The molecular method has made a new era in the analysis of microbial communities. Carl Woese started the concept of molecular analysis on microbes in the 1970s. He used rRNA as a molecular marker in classification analysis [22]. The use of sequencing at that time was conventional methods called Sanger [23]. The Sanger method is a sorting method that uses a single strand as a template. This method has the disadvantage of working for a long time and high running costs. Even the Sanger method will require approximately 15 years and cost the US $ 100 million to do the sequencing of the human genome [24].

The second-generation sequencing method emerged after researchers used the Sanger method for more than three decades. This method is often referred to as next generation sequencing (NGS). Several technology platforms included in the second-generation sequencing method are Roche/454, Ion torrent, and Ilumina [24]. According to Bragg & Tyson [25], second-generation sequencing has advantages over its predecessor, namely, (1) more efficient speed, (2) cheaper running costs, and (3) sequencing results that can be detected immediately without electrophoresis. Table 1 presents specific data on the different characteristics of the technology used in second-generation sequencing [23].

**Table 1:** Comparison of the characteristics in second-generation sequencing

Characteristics	Roche 454	Ion torrent	Illumina
Maximum read length (bp)	1200	400	300
One-way results (Gb)	1	2	1000
Amplification for library construction	Yes	Yes	Yes
Cost/Gb ($)	9538.46	460.00	29.30
Error rate (%)	1	~ 1	~ 0.1
Running time (H)	20	7.3	144

The second generation of sequencing technology that has been sufficiently developed still has problems regarding costs, results, and time that might be optimized again. Those problems lead to the development of third generation of sequencing technology. Third-generation sequencing has advantages over the second generation, namely, lower sequencing costs, no PCR processing, and a faster process [24]. The technology platforms included in the third-generation sequencing method are PacBio RS (Pacific Bioscience) and Oxford Nanopore [23].

### Basic methods in metagenomics analysis

Method selection is an essential strategy in the metagenomics analysis. In summary, the method is divided into two, namely, the molecular and bioinformatic methods [26].

#### Molecular method

Metagenomics is the study about the genome of the environmental community (metagenome) as the subject of research. This study is slightly different from genome studies focused on an individual (single genome).

##### Metagenomic DNA extraction

The extraction of DNA metagenome is carried out directly from environmental samples. This process is the first step in accessing the DNA metagenome. Some researchers use different methods, depending on the type of research sample used [10]. Tanveer et al. [27] have carried out DNA extraction of the metagenome using commercial kits and standard protocols.

Metagenomic DNA extraction using commercial kits is the easiest method because it only uses chemicals that have been provided by the manufacturer. According to Lear et al. [28], some researchers use branded kits based on the type of sample to be extracted. The PowerSoil and DNeasy PowerMax (Qiagen) kits are the most popular kits for researchers in soil samples, while the DNeasy Blood and Tissue Kits (Qiagen) kits are the most commonly used kits for seawater and groundwater samples.

In contrast to commercial kits, the use of standard protocols takes longer time than commercial kits [29]. Therefore, researchers prefer kits because they are more efficient in terms of time. However, some studies that use standard protocols show better results when compared to kits. Tanveer et al. [27] tried to compare the extraction of metagenomic DNA from the soil using the HiPurA soil DNA isolation kit (Himedia) and standard protocol. The results revealed that the standard protocol produced the highest concentration of DNA. Hassan et al. [30, 31] have also proven that the use of standard protocols produces higher concentrations than the DNA isolation kit for water (Epicenter).

Metagenomic DNA extraction is a crucial process because it will have an impact on the success of the further stage. According to Felczykowska et al*.* [32], the extraction of metagenome must produce a perfect DNA size. The size of fragments typically used for metagenome analysis is 600 bp to 25 kbp. Poor results will make the extracted sample unusable for further metagenomic analysis. Therefore, it is necessary to pay attention to the following: (1) do not physically interfere with genetic material and (2) contamination with protein, humic acid, and metals must be avoided. Other factors that might affect the results of DNA extraction are pH, soil mineral level, and soil type [33].

##### Calculation of concentration and purity of metagenomic DNA extracts

Determination of DNA concentrations and purity values can be calculated through 3 methods, namely, UV absorbance, fluorescent staining, and diphenylamine reaction [34]. The UV absorbance method is the most popular method for researchers to calculate the concentration and purity of DNA. It is because the UV absorbance method is easy, practical, and inexpensive [35].

Calculating the concentration and purity of DNA requires a device known as a spectrophotometer [35]. The principle of the UV absorbance ray method is the utilization of specific wavelengths that can be captured by DNA molecules [34]. DNA has the highest UV absorption at a wavelength of 260 nm, while proteins at a wavelength of 280 nm. Therefore, the wavelength ratio used when calculating the purity of nucleic acids is A260/A280. DNA samples have a purity ratio of around 1.8–2.0 [35]. The ratio value 260/230 can be used to help evaluate the presence of salt compounds, proteins, guanidine HCL, EDTA, lipids, and phenols. The lower the value, the higher the number of contaminants [36].

Contaminants can worsen DNA purity results. The most common contaminants in metagenome samples are humic acid and protein [32]. Protein and phenol contaminants usually show absorption values of 260/280, which are lower than 1.6. Meanwhile, if the absorption ratio value of 260/280 is more than 2.0, it indicates the presence of RNA contamination to DNA [36].

##### Gel Electrophoresis

Gel electrophoresis is a standard qualitative method used to separate, identify sizes, and purify nucleic acids. This method uses a gel media that has pores and can be passed through by nucleic acids [37]. Nucleic acids have phosphate groups that make these molecules negatively charged so that nucleic acid molecules will move towards the anode (positive electrode) when energized. The speed of this transfer is influenced by the factor of molecular weight, gel concentration, and the electrical voltage used [38].

Agarose gels are the most popular in gel electrophoresis. Agarose gels are polymers consisting of disaccharide units, which are arranged repeatedly and consist of galactose and 3,6-anhydrogalactose. This gel is made from seaweed extract and has large pores [37]. Pore size can be affected by gel concentration. Each gel concentration profile shows the optimal state of the length of the nucleic acid fragment used as a sample when running gel electrophoresis. Gutiérrez-lucas et al*.* [39] have used a 0.8% gel concentration for samples originating from the soil. The choice of 0.8% agarose gel concentration is a strategy for electrophoresis from metagenomic samples because environmental DNA fragments (eDNA) have an extended size. Table 2 presents recommendations for gel concentrations used and adjusted based on the length of the nucleotide acid fragments used for the sample [38]:

**Table 2:** Recommended agarose gel concentrations based on fragment length from nucleic acid samples

Gel concentration (%)	The size of nucleic acids (kb)
0.3	5–60
0.6	1–20
0.7	0.8–10
0.8	1–7
0.9	0.5–7
1.2	0.4–6
1.5	0.2–3
2.0	0.1–2

##### Amplification of 16S rRNA gene

Ribosomes are essential compounds for protein synthesis. They are very conservative and often used as a standard for determining taxonomies. Prokaryotic microbes are generally composed of 65% rRNA (ribosome-ribonucleic Acid) and 35% protein. Each prokaryotic ribosome consists of 2 subunits, namely, large subunits (LSU) (the 50S), which contain two rRNA molecules (5S and 23S) and small subunits (SSU) (30S) that contain a single rRNA molecule (16S) [40].

16S rRNA is an area often used as a standard for taxonomy profiling analysis in prokaryotic organisms [41]. This gene has nine regions called hypervariable regions (V1-V9) with a total length of about 1500 bp. These nine regions can distinguish the diversity of prokaryotic organisms [40, 42]. There are three reasons for 16 rRNAs as an appropriate marker for taxonomy profiling, and these are (1) the 16 rRNA genes that are present in all prokaryotic organisms; (2) it is almost impossible to experience lateral gene transfer; and (3) the conservative ribosomal protein structure makes the sequence very sustainable [40].

The identity and frequency of microorganisms can be seen by reading 16S rRNA sequences using sequence homology. Readings of genus and species identities can usually be distinguished at a minimum level of 95% for the genus and 97% for species; whereas for strain levels, it is distinguished at a minimum level of 99% [43]. Generally, the V2-V3 region is an excellent area to be used as a gene marker in metagenomic studies. However, several researchers have used various target areas in the V region of the 16S rRNA gene in the analysis of the diversity of microorganisms. According to Zhang et al*.* [44], the use of different target areas V will result in different bacterial community compositions. The best results were found using the V1-V2 and V1-V3 regions.

#### Bioinformatics method

The bioinformatics plays a vital role in the metagenomics analysis. Niu et al*.* [45] explain the role of bioinformatics in metagenomic analysis, for example, as in the analysis of 16S rRNA data. Analysis of 16S rRNA data can be used to determine the diversity of samples and predict the metabolic pathways of microbes in the sample. An example of a tool used for diversity analysis is MOTHUR. Mallick et al*.* [46] have reported the use of 16S rRNA sequence data to predict the metabolic pathway of a community from the sample used using the PICRUSt software.

The use of bioinformatics tools is based on objectives in exploration. Bioinformatic tools help to analyze environmental samples. Several types of bioinformatics analysis in metagenomics approaches are:

1. Assembly

Assembly is a process of reconstructing short metagenome reads joined to form a long sequence. The long sequence is called as contigs [3]. Assembly uses one of two methods that are often used, OLC and the de Bruijn graph [14]. In addition, other researchers have also developed assembly methods such as hybrid and Iterative joining [47, 48]. However, the de Bruijn graph is the most popular method. The advantage of the de Bruijn graph is cheaper than OLC because it can be built without pairwise comparisons [14]. Bioinformatic tools that can be used in assembly are BBAP, Genovo, MegaGT, and MEGAHIT [49].

2. Binning

Binning is the clustering process of sequences that have been constructed in the assembly process. Binning groups sequences called contigs into classes, so they represent a biological taxon [14]. This method is carried out after assembling raw sequences reads into contigs [50]. Some software options used for binning analysis are MetaWatt [50] and CONCOCT [51]. MetaWatt has advantages that are higher accuracy than existing methods and easy to use [50]. While CONCOCT has reported by the author, this software has high precision and can group complicated microbial communities [51].

3. Sequence analysis

Sequence analysis is a method of finding parts of the same biological sequence [52]. Sequence analysis is divided into two ways, namely, simple alignment and multiple alignments. Simple alignment is the alignment between two sequences, while multiple alignments are the alignment of more than two sequences [53]. One of the tools used for alignment is BLAST (Basic Local Alignment Tool). BLAST is a tool used to compare sequences of various types of organisms. The score of each alignment is given an expectation value (*E* value), which is a measure of statistical significance [54].

4. Pfam analysis

Pfam is a database of protein families. Pfam’s analysis refers to the double alignment produced using the hidden Markov model. The purpose of Pfam’s analysis is to look at the relationship between protein sequences at the family level [54].

5. Analysis of protein structure prediction

The sequence of amino acids is called the primary structure. The primary structure comes from the sequence of the genes that encode it. The structure of proteins is classified as secondary, tertiary, and quaternary structures. Knowledge of the protein structure is fundamental in understanding the function of proteins. Prediction analysis of protein structure by bioinformatics can help in understanding the physical characteristics of a protein and its functions [52].

6. Phylogenetic analysis

Phylogenetic analysis of functional metagenomics refers to procedures used to reconstruct the evolutionary relationships between groups of protein molecules and to predict certain features of a molecule. The methods for forming phylogenetic trees are likelihood methods, parsimony methods, and distance methods. There is no perfect method, and each has specific strengths and weaknesses. The example tools used in phylogenetic analysis are MEGA (Molecular Evolutionary Genetics Analysis), MOLPHY, and PHYLIP [55].

### Exploration of novel enzymes with the metagenomic approach

The effort to explore natural resources is a strategy in optimizing the use of genetic resources. Enzymes are one of the compounds explored from nature to be taken advantage of in the industrial field. These biocatalysts are not only crucial for cell biochemical processes but also in today’s modern industry application. Robinson [2] added that enzymes could be useful in the pharmaceutical industry for modifying antibiotics, the soap industry, and also for the benefit of forensic and clinical testing.

Exploration of enzymes using a metagenomic approach is not something new. In 1985, Pace and colleagues introduced direct cloning from environmental samples [16]. The first study of screening based on functional genes was successfully conducted by Healy et al*.* [56], who reported on the isolation of functional genes that encode cellulase enzymes from the environment. Five years later, Rondon et al*.* [57] have used Bacterial Artificial Chromosome (BAC) as a vector to create a metagenome library from soil samples. Some enzymes are found by Rondon et al*.* [57], namely, lipases, amylases, and nucleases.

### Recent research on exploration of novel enzymes with metagenomic approach

Research on enzyme exploration in the past still used conventional methods by culturing the microorganisms on the growth media. However, the development of technology currently directs researchers to the exploration of novel enzymes without culturing on growth media. Cellulase, lipase, and protease enzymes are types of enzymes that are important for industrial processes [58].

#### **Cellulases**

Cellulases are a group of enzymes catalyzing cellulose polymers into simpler sugars [59]. This enzyme is useful for the paper industry, cotton processing, and detergents [60]. Exploration of cellulase enzymes in a conventional way has placed *Aspergillus* sp. as an organism that has high cellulase activity [61]. However, metagenomic methods reveal that cellulase enzymes can be found widely in various types of organisms. Cui et al*.* [17] reported that organisms such as *Cloacibacterium, Paludibacter, Exiguobacterium, Acetivibrio, Tolumonas*, and *Clostridium* are known to be cellulolytic microbes and have the potential to produce cellulase enzymes. These six genera were found in high cellulose environments in bamboo paper making plants.

Previous research revealed that the genes encoding the cellulase enzyme were also found in the human intestinal microbial community [6] and the microbial community of bagasse waste [7]. Currently, cellulase enzyme exploration also leads to high-temperature environments such as hot springs [62]. The selection of extreme environments is carried out with the hope of getting enzymes with high temperature (thermostable) resistance characteristics.

#### **Proteases**

Proteases are enzymes that hydrolyze peptide bonds in amino acid chains. This enzyme is used in the detergent, pharmaceutical, and food and beverage industries [63]. Protease sources are spread in several organisms, such as plants, animals, and microorganisms. Currently, the best-known protease producer in the industry is *Bacillus* sp. [64]. The development of metagenomic technology enables the search for other organisms that are potentially more efficient in terms of effectiveness. Biver et al. [18] reported the discovery of a new protease-coding gene derived from a microbe similar to *Desulfobacter postgatei* 2 ac9 with a similarity rate of 69%. Also, Devi et al*.* [65] reported the findings of the Prt1A gene that encodes the protease enzyme from organic sludge. The protease enzyme from the Prt1A gene is known to be optimal at 55 °C. The following year, Pessoa et al*.* [66] discovered a gene that codes for proteases with optimum activity at 60 °C.

#### **Lipases**

Lipases are enzymes that catalyze the hydrolytic cleavage of the ester bonds between carboxylic acids and alcohol groups [67]. This enzyme is used in the detergent, food, biodiesel, and bioremediation industries. *Bacillus* spp. bacteria such as *B. alcalophilus*, *B. licheniformis*, *B. pumilus*, and *B. subtilis* are the most well-known producers of bacterial lipases at present [30, 31, 68].

Researchers are currently competing in exploring other organisms that have the potential to produce better lipase. Hardeman & Sjoling [69], with a functional metagenomic approach, have found the h1Lip1 gene that has a similarity to the lipase of *Pseudomonas putida* with a similarity level of 54%. Lipase enzyme is from the optimum h1Lip1 gene at 35 °C (low temperature). According to López-lópez et al. [67], the maximum lipase character at low temperatures is generally suitable for the cold washing process in detergents. Besides, many other enzymes sourced from the metagenome library have unique biochemical properties that make them valuable for industrial applications. An example is an enzyme that is resistant to solvents, and detergents found in soils are contaminated with petroleum hydrocarbons [19].

#### Other enzymes

There are many enzymes found by researchers from metagenome-source, which can potentially be commercialized. Recently, Sharma et al. [70] have reported novel bleomycin resistance dioxygenase (BRPD) from contaminated agricultural soil. It has a function in the bioremediation process by catalyzing the degradation of hydrocarbon substrate like pesticides. In addition, Berini et al. [71] also have found *53D1* gene encoding chitinases which can potentially be used for controlling plant pests. They investigated chitinases controlling in *Bombyx mori,* a Lepidoptera. The result showed that chitinase (*53D1* gene) was a promising enzyme used as an insecticide. Other recent studies have also revealed enzymes derived from metagenome sources, such as oxoflavin-degrading enzyme used in the agricultural industry [72], transaminases used in the pharmaceutical industry [73], and AHL-lactonase [74].

#### The patented enzymes from metagenome-source for industry

In the past 5 years, several patented enzymes beneficial to the industry are published. Previously, the patented enzymes for commercialization are reported by Berini et al. [75]. This review presents novel patented enzymes published in the past 5 years (Table 3). The lists patented enzymes from metagenome source include cellulases, protease, lipase, α-amylase, chitinase, β-glucosidase, and endoglucanase.

**Table 3:** Examples of patented enzymes from metagenome source in the past 5 years

Patent Number	Country	Assignee	Enzyme	Source	Year	Application
CN108463551A	China	Scientific and Industrial Research Council	Cellulases	Soil	2016	food and feed industry, detergent, weaving, and biofuel industry
BR102016000771A2	Brazil	Universidade Estadualhed De Santa Cruz	Proteases	mangrove sediment	2016	antitumor, antifungal, antiviral, and antiparasitic treatment
KR102026836B1	South Korea	Korea Research Institute of Chemical Technology	Lipase	Soil	2018	industrial mass-production, biopharmaceuticals, and biodiesel
KR101816615B1	South Korea	Republic of Korea	α-Amylase	black goat rumen	2015	feed additive, detergents, and biofuel
CN107475273B	China	Chengdu Institute of Biology, Chinese Academy of Sciences	Chitinase	Wetland environment	2017	the industries of food, medicine, agriculture, and cosmetics
CN107828806A	China	Guangdong Pharmaceutical University	β-glucosidase	Soil	2017	The industry of pharmaceuticals, food, bioethanol, and medicine
JP6552098B2	Japan	Honda Motor Co., Ltd., Kazusa DNA Research Institute	Endoglucanase	Hot spring soil	2016	bioethanol

#### Challenges in exploration of novel enzymes with the metagenomic approach

The selection of sampling locations is a challenge for researchers to explore novel enzymes. A location will determine the role of enzyme characterization. Each location has a unique ecological niche for the exploration of novel enzymes. Unique niches are created by functional interactions between the microbial community and their environment [76, 77]. Mhuantong et al*.* [76] reported the discovery of high cellulolytic bacteria in sugarcane bagasse samples. The number of cellulolytic bacteria even looks significant when compared to in-cellulolytic bacteria. Nie et al*.* [78] also have reported that the microbial community in the oil environment had genes that encoded hydrocarbon degradation enzymes. The existence of hydrocarbon degradation enzymes can not be separated from the oil environment rich in hydrocarbon compounds. The research evidence reinforces the theory that the environment determines the characterization of enzymes, so the consideration of selecting the sampling location needs to be adjusted to the type of enzyme to be explored. Also, the characteristics of sampling locations pose challenges for researchers. Locations with extreme characters require special techniques and caution in sampling, for example, sampling at hot spring locations [79].

The choice of DNA extraction methods from environmental samples is also a challenge because it has greater difficulty than DNA extraction from a single genome. Difficulties arise due to DNA from the environment that contains more contaminants, such as humic acid, protein, and carbohydrates. In addition, if clay sample is used, it will be more difficult to extract the DNA as it is bound to soil particles [80]. Therefore, researchers need to do the special treatment of samples that have unique characteristics.

## Conclusions

Metagenomics is the study of genomes from microorganism communities in the environment. Metagenomics is divided into two primary studies, namely, structural and functional metagenomics. Basic structural metagenomics method consists of assembly, binning, and microbial community analysis such as taxonomic profiling, gene prediction, and metabolic pathways. Meanwhile, functional metagenomics approach consists of gene construction, screening, heterologous overexpression, bioinformatic analysis, and protein product characterization.

Exploration of novel enzymes with a metagenomic approach has revealed several novel enzymes from nature, such as cellulases, proteases, lipases, and other enzymes such as BRPD, chitinases, oxoflavin-degrading enzyme, transaminases, and AHL-lactonase. The existence of metagenomics has helped researchers uncover novel enzymes from nature that are beneficial to the industries. Understanding of metagenomic and its application is expected to have an impact on the development of technology that is useful for humanity.

## Data Availability

Not applicable

## Abbreviations

eDNA: Environmental DNA
NGS: Next Generation Sequencing
bp: Base pair
kb: Kilobase pair
Gb: Gigabase pair
rRNA: Ribosome-Ribonucleic Acid
LSU: Large Subunits
SSU: Small Subunits
BLAST: Basic Local Alignment Tool
MEGA: Molecular Evolutionary Genetics Analysis
BAC: Bacterial Artificial Chromosome
